# The impact of primary immunization route on the outcome of infection with SARS-CoV-2 in a hamster model of COVID-19

**DOI:** 10.3389/fmicb.2023.1212179

**Published:** 2023-05-24

**Authors:** Edward G. Barrett, David Revelli, Chandra Shekhar Bakshi, Alan Kadish, Salomon Amar

**Affiliations:** ^1^Lovelace Biomedical Research Institute, Albuquerque, NM, United States; ^2^Department of Pathology, Microbiology and Immunology, New York Medical College, Valhalla, NY, United States; ^3^Touro University, New York City, NY, United States; ^4^New York Medical College, Valhalla, NY, United States

**Keywords:** COVID-19, SARS-CoV-2, hamsters, intranasal vaccination, subcutaneous vaccination, immune response

## Abstract

The COVID-19 pandemic, caused by the SARS-CoV-2 virus, has resulted in over 6.7 million deaths worldwide. COVID-19 vaccines administered parenterally via intramuscular or subcutaneous (SC) routes have reduced the severity of respiratory infections, hospitalization rates, and overall mortality. However, there is a growing interest in developing mucosally delivered vaccines to further enhance the ease and durability of vaccination. This study compared the immune response in hamsters immunized with live SARS-CoV-2 virus via SC or intranasal (IN) routes and assessed the outcome of a subsequent IN SARS-CoV-2 challenge. Results showed that SC-immunized hamsters elicited a dose-dependent neutralizing antibody response but of a significantly lower magnitude than that observed in IN-immunized hamsters. The IN challenge with SARS-CoV-2 in SC-immunized hamsters resulted in body weight loss, increased viral load, and lung pathology than that observed in IN-immunized and IN-challenged counterparts. These results demonstrate that while SC immunization renders some degree of protection, IN immunization induces a stronger immune response and better protection against respiratory SARS-CoV-2 infection. Overall, this study provides evidence that the route of primary immunization plays a critical role in determining the severity of a subsequent respiratory infection caused by SARS-CoV-2. Furthermore, the findings suggest that IN route of immunization may be a more effective option for COVID-19 vaccines than the currently used parenteral routes. Understanding the immune response to SARS-CoV-2 elicited via different immunization routes may help guide more effective and long-lasting vaccination strategies.

## Introduction

More than 2 years since the first COVID-19 case was reported in the United States, the pandemic caused by the SARS-CoV-2 virus continues. In the past 2 years, the official death toll has crossed over 1 million in the United States. Even with the availability of several vaccines, thousands of cases are still reported weekly in the United States alone, indicating an urgent need for improved vaccination strategies focused on enhancing immunogenicity and the duration of protection. Given the guidelines provided by the Centers for Disease Control and Prevention and the availability of vaccines, COVID-19 cases were expected to subside rapidly, but on the contrary, the pandemic continues. While some argue that the pandemic’s persistence stems from the political climate and vaccination hesitancy, the fact remains that current vaccines have their limitations. The technical factors limiting accessibility to vaccines center on the requirements for the storage of these vaccines at an ultra-low temperature to maintain their stability. This has not only been challenging in wealthy countries like the United States but is more problematic in lower-income countries, leaving communities vulnerable to continued widespread SARS-CoV-2 transmission (Johnson et al., 2022). Further, the currently approved COVID-19 vaccines in the United States are administered parenterally through intramuscular (IM) or subcutaneous (SC) injections. It has been well established that the parenteral route of administration does not induce an effective mucosal immune response required for the protection of mucosal surfaces of the upper respiratory tract where respiratory viruses like SARS-CoV-2 first enter the body (Park and Lee, 2021). Another challenge is that the IM injections of the current mRNA-based vaccines require administration by healthcare professionals (Nguyen et al., 2022).

Given that the upper respiratory tract also provides the first line of defense against respiratory pathogens, it is prudent to further analyze the viability of intranasal (IN) immunization as a potential platform for overcoming many of the challenges associated with the route of administration of current COVID-19 vaccine (Johnson et al., 2022). IN immunization has been proposed as a potential way to offer stronger and more durable protection against pulmonary infectious diseases, such as SARS-CoV-2, with encouraging findings indicating a reduced risk of breakthrough infections (Tiboni et al., 2021; Chiuppesi et al., 2022; Nguyen et al., 2022; Nouailles et al., 2023). The current study used Syrian hamsters to compare immune responses and protective efficacy induced by immunization via SC and intranasal (IN) routes, followed by an IN challenge. We employed homologous and heterologous immunization and challenge model by utilizing SARS-CoV-2 isolate USA-WA1/2020 for immunization and challenge studies. We report that the route of primary immunization plays a critical role in determining the severity of a subsequent respiratory infection caused by SARS-CoV-2. The current study supports the idea that IN immunization develops a reliably strong protective immune response to live SARS-CoV-2 and supports a shift to IN immunization strategies.

## Materials and methods

### SARS-CoV-2 propagation

SARS-CoV-2 isolate USA-WA1/2020 was obtained from the World Reference Center for Emerging Viruses and Arboviruses. The virus was passaged once in Vero E6 AGM kidney cells (BEI Resources) at the University of Texas Medical Branch, Galveston. The virus was stored at −70°C or below until used. The virus was thawed for immunization and challenge studies, diluted with Dulbecco’s Modified Eagle’s Medium (DMEM) to about 1 × 10^6^ TCID_50_/mL, and stored on ice until the inoculations were done. Two aliquots of each inoculum were used for back titration and confirmation of the inoculation dose by TCID_50_ assay using Vero E6 cells. The SARS-CoV-2 virus used was at the fifth passage from the original patient isolate.

### Hamster immunization and challenge studies

All immunization and challenge studies were performed in an animal biosafety level 3 (ABSL3) facility at the Lovelace Biomedical Research Institute (LBRI), Albuquerque, New Mexico. All animal studies were conducted according to the protocols approved by the Institutional Animal Care and Use Committee (IACUC). Male Syrian hamsters (*Mesocricetus auratus*), 6–10 weeks old and with an average body weight of 114.96 g ± 5.22 grams (mean ± SD) were used. Animals were individually housed in micro-isolator cages. During handling, individual cages were disinfected inside a biosafety hood, and all precautions were taken to reduce the risk of potential cross-contamination between the cages. The sham control group was initially housed outside the ABSL3 facility to avoid cross-transmission during the initial SC and IN immunizations. The hamsters in the sham control group were brought into the ABSL3 facility prior to the SARS-CoV-2 challenge. The group size of five animals per group was chosen based on the published literature (Roberts et al., 2005; Chan et al., 2020).

#### Immunization

**Figure 1:**
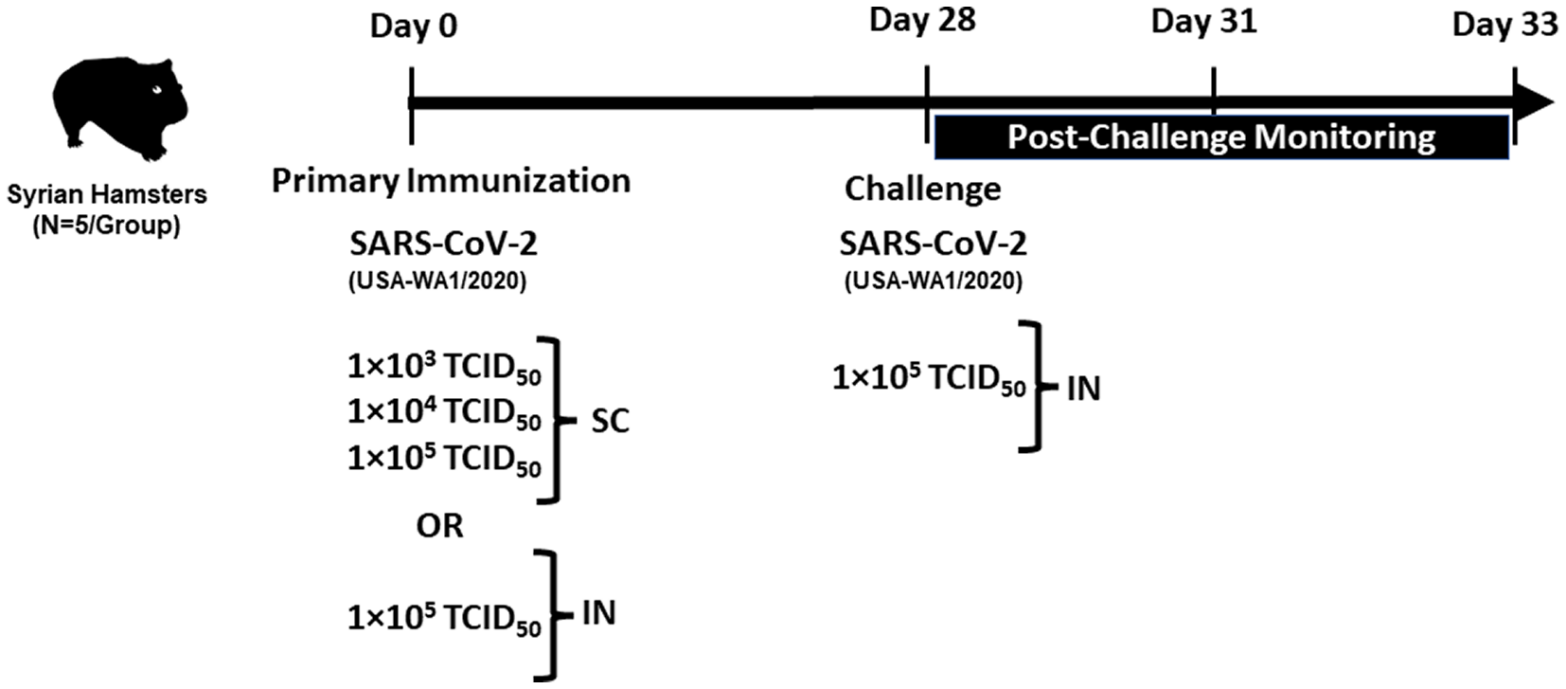
The plan for immunization and challenge studies.

The overall plan for immunization and challenge studies is shown in Figure 1. All SC injections were administered in the neck. Hamsters (*n* = 5 per group) were given increasing doses of 1 × 10^3^, 1 × 10^4^, and 1 × 10^5^ TCID_50_ of SARS-CoV-2 by SC injection in a volume of 200 μL/animal. An additional group of hamsters (*n* = 5) received a target dose of 1 × 10^5^ TCID_50_ by IN instillation (100 μL/nares, 200 μL/animal). The sham control group was administered sterile saline (200 μL/animal) by SC injection.

#### Challenge

On day 28 post-primary immunization, sham control, and SC or IN-immunized animals were challenged IN with a target dose of 1 × 10^5^ TCID_50_ of SARS-CoV-2 (100 μL/nares, 200 μL/animal).

Before IN immunization and challenge, all animals were anesthetized with a cocktail of ketamine and xylazine (80 mg/kg and 5 mg/kg, respectively) administered intraperitoneally. All animals were observed twice daily after immunization and challenge for clinical signs and morbidity by recording body weights throughout the study. All animals were humanely euthanized on day 33 post-primary immunization (5 days post-challenge), and lungs were collected to quantify viral load and for histopathological studies.

### Sample collection and processing

On day 28, before the IN SARS-CoV-2 challenge, blood was collected from all animals, and serum was separated for neutralizing antibody determination. Animals had their nasal passages swabbed on day 3 post-challenge (day 31 post-primary immunization) for analysis by qRT-PCR. For collecting nasal swabs, animals were sedated with isoflurane or by ketamine/xylazine (80 mg/kg; 5 mg/kg; intraperitoneally), after which 0.5 mm diameter Ultrafine Micro Plasdent swabs, or equivalent, were inserted approximately 1–3 mm deep into the nasal cavity to collect samples. On day 5 post-challenge (day 33 post-primary immunization), animals were euthanized by intraperitoneal injection with an overdose of a barbiturate-based sedative. Terminal body weights and lung weights with trachea were recorded. Portions of the lung (left lobe, two separate samples, weighed) were harvested for qRT-PCR and TCID_50_ analyses. In addition, the right lung lobes were infused with 10% neutral-buffered formalin, trimmed, paraffin-embedded, sectioned at 4 μm, and stained with hematoxylin and eosin for histological evaluations.

### Quantitation of SARS-CoV-2 by qRT-PCR

SARS-CoV-2 viral RNA was quantified by a qRT-PCR assay targeting the SARS-CoV-2 nucleocapsid phosphoprotein gene (N gene). Lungs were homogenized in Trizol using a TissueLyser and centrifuged at 4000 × *g* for 5 min. From the supernatants, RNA was isolated using the Direct Zol-96 RNA Kit (Zymo Research), according to the manufacturer’s instructions. qRT-PCR was performed using the SARS-CoV-2N gene forward primer 5′TTACAAACATTGGCCGCAAA3′; reverse primer 5′GCGCGACATTCCGAAGAA3′ and SARS-CoV-2 probe 6FAM-ACAATTTGCCCCCAGCGCTTCAG-BHQ-1. Amplification and detection were performed using a real-time thermal cycler under the following cycling conditions: 50°C for 5 min, 95°C for 20 s and 40 cycles of 95°C for 3 s, and 60°C for 30 s. Genome copies per gram of tissues were calculated from a standard curve generated from RNA standards of known copy concentration. All RNA samples were run in triplicate.

SARS-CoV-2 subgenomic copies were quantified by qRT-PCR targeting the SARS-CoV-2 E gene, and genome copies per milliliter or gram equivalents were calculated from a standard curve. The SARS-CoV-2 E gene sgLead SARS-CoV-2 forward primer 5′-CGATCTCTTGTAGATCTGTTCTC-3′; Sarbeco reverse primer 5′-ATATTGCAGCAGTACGCACACA-3′; and E Sarbeco probe, 6FAM-ACACTAGCCATCCTTACTGCGCTTCG-BHQ-1 were used for amplification. Amplification and detection were performed under the following thermal cycling conditions: 50°C for 5 min, 95°C for 20 s, 40 cycles of 95°C for 3 s, and 60°C for 30 s. All RNA samples were run in triplicate.

### Determination of infectious viral titers

Infectious virus titers were determined in a TCID_50_ assay using Vero E6 cells in a 96-well format. Vero E6 cells were plated on flat-bottom 96-well tissue culture plates to ≥ 90% confluency. Ten-fold serial dilutions of each sample were prepared in viral infection media (VIM) containing DMEM, 2% fetal bovine serum and 1% penicillin/streptomycin. Diluted samples were added (100 μL/well), with five replicates of each. Plates were incubated at 37°C for about 72 h or until the cytopathic effect (CPE) was discernable. Stock virus of known concentration and blank VIM served as positive and negative controls, respectively. At assay completion, cells were fixed with 50 μL of 4% formalin per well for a minimum of 2 h at 2–8°C. The fixed cells were stained with 10 to 20 μL of crystal violet per well for at least 1 h at room temperature. CPE was visually assessed for each well, and the TCID_50_ titer was calculated according to the Reed-Muench method.

### Histopathology

Hematoxylin and eosin-stained sections of the right lung lobes were evaluated. Transverse sections of the right cranial and accessory lung lobes, and longitudinal sections of the right middle and caudal lobes were examined. The sections were randomized for the order in which their lung sections were read. The slides were scored for the extent of peribronchiolitis, perivasculitis, interstitial inflammatory cell infiltrates, and alveolitis. In each of these inflammatory categories, the severity of the infiltration of mononuclear leukocytes was graded along with the infiltration of granulocytes. Severity was subjectively scored on a 5-point scale (0 = absent, 1 = minimal, 2 = mild, 3 = moderate, and 4 = marked). To determine the extent of the lesions, a distribution score on a six-point scale was also given to each of the findings (0 = absent, 1 = focal, 2 = locally extensive, 3 = multifocal, 4 = multifocal and coalescing, and 5 = diffuse). Other findings were also graded, including mucous cell hyperplasia/hypertrophy and bronchiolar epithelial hyperplasia.

### Neutralization assay

Titers of neutralizing antibodies to SARS-CoV-2 were determined in serum samples by micro-neutralization assay using Vero E6 cells in 96-well plates. Serum samples were heat-inactivated at 56°C for 30 min and subsequently serially diluted 2-fold, covering appropriate ranges for each experimental group. SARS-CoV-2 (strain USA-WA1/202) stock was diluted to reach a concentration of 100 TCID_50_/25 mL, and then equal volumes of serum dilution and virus were mixed and incubated for 1 h at 37°C in a humidified atmosphere with 5% CO_2_. After incubation, 50 mL of each neutralization mixture was dispensed in six technical replicates into wells containing a semi-confluent Vero E6 cell monolayer in a 96-well plate. Plates were incubated for 1 h at 37°C in a humidified atmosphere with 5% CO_2_. Wells were then replenished with 100 mL of infection medium and incubated at 37°C and 5% CO_2_ for 3 days. At assay completion, cells were fixed with formalin and stained with crystal violet for visualization of the cytopathic effect. Fifty percent neutralization endpoints were calculated using the Reed and Muench method.

### Determination of SARS-CoV-2 spike protein-specific IgG antibodies

As reported previously (Johnson et al., 2022), briefly, microtiter plates (Nunc MaxiSorp) were coated with 1 μg/mL of SARS-CoV-2 S protein (GenScript) in carbonate buffer (pH 9.4) and incubated overnight at 4°C before blocking with 100 μL PBS-0.05% Tween (PBST) containing 1% bovine serum albumin (BSA) for 1 h. Serum samples were serially diluted in PBST. After 2 h at room temperature, the plates were washed three times with PBST, then 100 μL per well of 1:3000 goat antihamster IgG-horseradish peroxidase (HRP; Thermo Fisher) in PBST with 1% BSA was added. After 1 h at room temperature, 50 μL/well of 3,3′,5,5′-Tetramethylbenzidine (TMB) substrate (Rockland) was added after three washes with PBST. After 10 min of development, 50 μL/well of 2 M sulfuric acid was used to stop the reaction. Optical densities (ODs) were measured at 450 nm with a Spectra Max M2 microplate reader.

### Statistical analyses

Data are expressed as mean ± standard error of the mean (SEM). Changes in body weights over time were evaluated utilizing a two-way repeated measures analysis of variance. IgG, nasal-lung genomic/subgenomic, and lung TCID_50_ results were tested for normality, and much of the data was not normally distributed. Thus, it was log-transformed for statistical analysis. All comparisons were made between SC and IN-immunized groups as well as the control group. All subgroup comparisons were performed using one-way analysis of variance using post-hoc Tukey’s test. *p* value was set at *p* < 0.05.

## Results

### IN-immunized hamsters are better protected than hamsters immunized with higher SC doses of SARS-CoV-2

**Figure 2:**
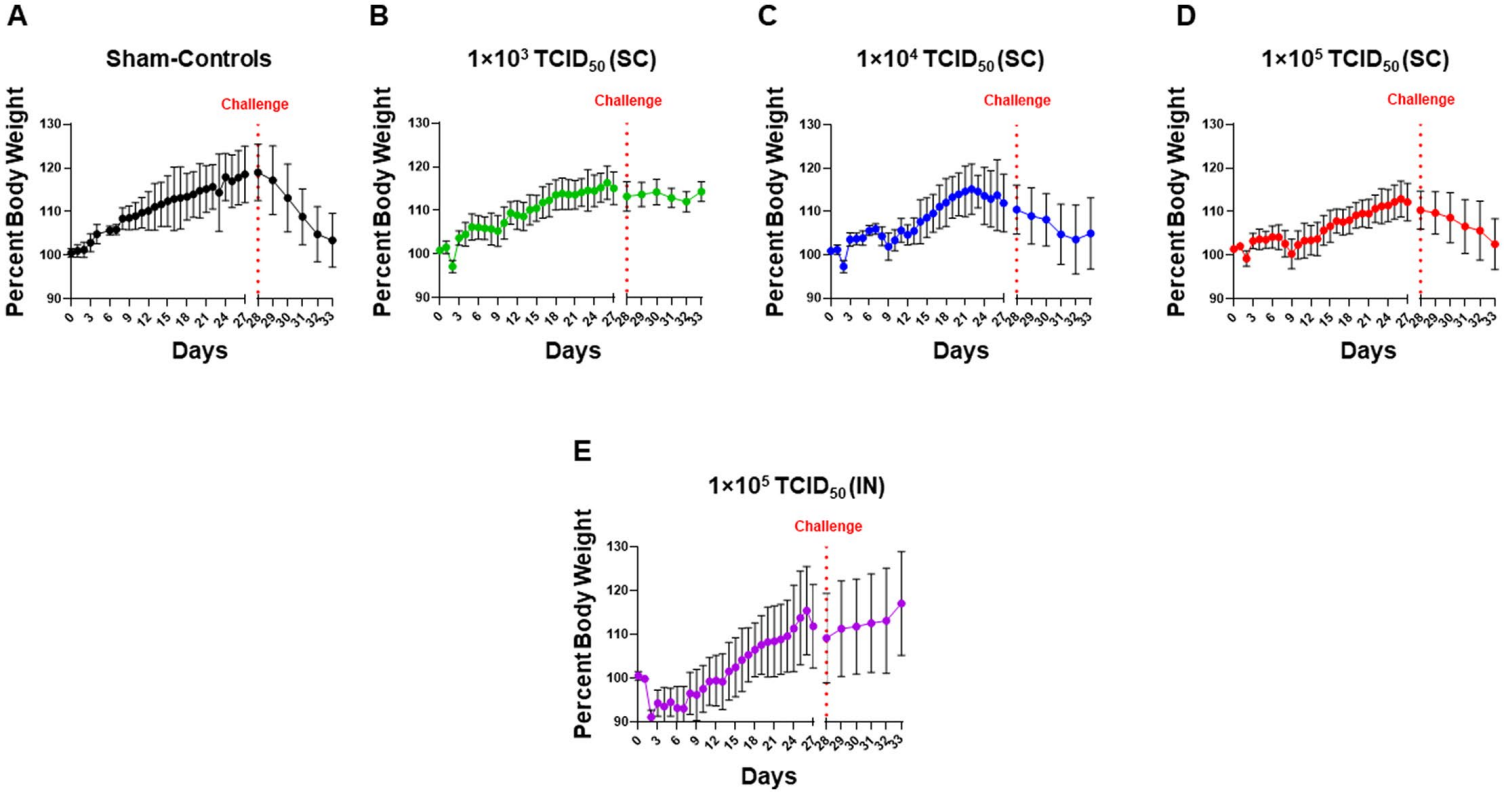
Intranasally-immunized hamsters are better protected than hamsters immunized with higher subcutaneous immunization doses of SARS-CoV-2. Syrian hamsters (*n* = 5 per group) administered with sterile saline subcutaneously (SC) **(A)** or were immunized with the indicated doses of live SARS-CoV-2 (USA-WA1/2020 isolate) by SC route **(B–D)** and intranasal (IN) route (**E**). Hamsters in all groups were challenged IN with 1 × 10^5^ TCID_50_ dose of live SARS-CoV-2 (USA-WA1/2020 isolate) on day 28 post-immunization as indicated by the vertical dotted red line. The pre- and post-challenge body weights of hamsters in all groups were recorded daily throughout the course of the experiment and plotted as mean ± SD of the percent body weights.

We monitored the body weights of hamsters immunized with increasing doses of SARS-CoV-2 via the SC route or a single dose via the IN route. Sham-inoculated hamsters served as controls (Figure 2A). In SC-immunized animals, there was a transient body weight loss for 2 days post-immunization, followed by a subsequent recovery of body weights. However, after the initial recovery, there was an apparent dose-dependent secondary drop on days 8–14 before recovering again (Figures 2B–D). Immunization with SARS-CoV-2 via the IN route resulted in an 8–10% body weight loss 2 days post-immunization and subsequent recovery (Figure 2E). Upon challenge with IN 1 × 10^5^ TCID_50_ of SARS-CoV-2 on day 28 post-immunization, the sham control group exhibited a more rapid weight loss (Figure 2A). The SC-immunized groups showed varied weight loss responses following the challenge. Minimal weight loss was observed in hamsters immunized SC with 1 × 10^3^ TCID_50_ (Figure 2B). A gradual weight loss was observed in hamsters immunized SC with 1 × 10^4^ TCID_50_ for 4 days post-challenge. These hamsters appeared to show recovery on day five post-challenge (day 33 post-immunization; Figure 2C). However, the group of hamsters immunized SC with 1 × 10^5^ TCID_50_ continued to lose body weight until day five post-challenge (day 33 post-immunization). Their pattern of weight loss mirrored that observed for the sham control hamsters (Figure 2D). On the contrary, hamsters immunized IN with 1 × 10^5^ TCID_50_ of SARS-CoV-2 did not lose weight post-challenge (Figure 2E). These results suggest that IN-immunized hamsters were better protected than their SC-immunized counterparts against an IN challenge with SARS-CoV-2, with the exception of those immunized SC with 1 × 10^3^ TCID_50_.

### Pre- and post-challenge neutralizing antibody response is significantly higher in IN-immunized than SC-immunized hamsters

**Figure 3:**
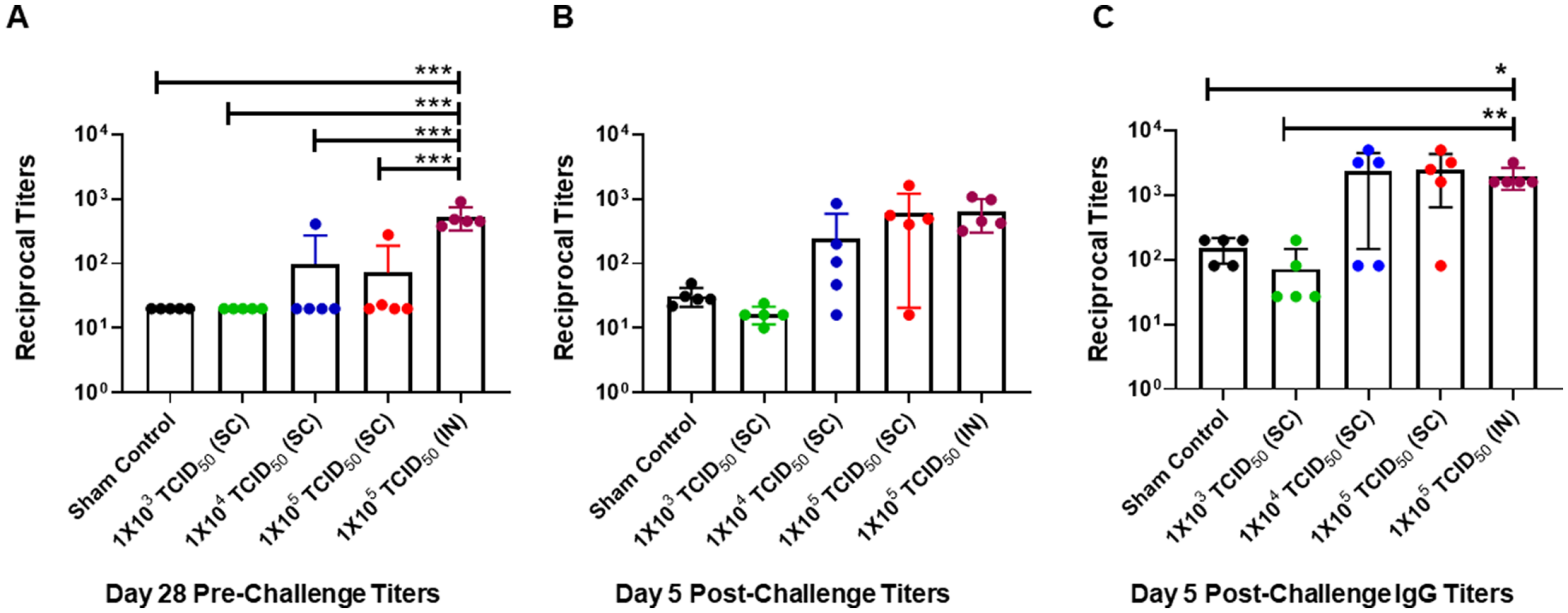
Pre- and post-challenge neutralizing antibody response is significantly higher in intranasally-immunized than subcutaneously immunized hamsters. **(A)** Pre-challenge serum neutralizing antibody titers in groups of hamsters immunized with the indicated doses of live SARS-CoV-2 by subcutaneous (SC) and intranasal (IN) routes were determined on day 28 post-immunization. Sham control hamsters were administered with sterile saline SC. **(B)** All groups were challenged with 1 × 10^5^ TCID_50_ dose of live SARS-CoV-2 (USA-WA1/2020 isolate) on day 28 post-immunization. The post-challenge serum neutralizing antibody titers **(B)** and SARS-CoV-2 spike protein-specific IgG titers were determined on day 5 post-challenge **(C)**. The results are expressed as reciprocal titers. The data were analyzed by ANOVA using post-hoc Tukey’s test. ^*^p < 0.05; ^**^p < 0.01; ^***^p < 0.001.

We assessed serum-neutralizing antibody responses in SC and IN-immunized hamsters before the challenge on day 28 post-immunization and on day five post-challenge (day 33 post-primary immunization). No pre-challenge SARS-CoV-2-specific antibody responses were observed in the sham control group, or the group of hamsters immunized SC with 1 × 10^3^ TCID_50_. Furthermore, only 1 in 5 hamsters from the groups immunized SC with 1 × 10^4^ or 1 × 10^5^ TCID_50_ showed slightly increased antibody responses compared to those observed for the sham control or the 1 × 10^3^ TCID_50_-immunized groups. However, all 5 hamsters from the IN-immunized group had significantly higher levels of SARS-CoV-2-specific antibodies than all of their SC-immunized counterparts on day 28 post-immunization (Figure 3A). These results indicate that, unlike SC immunizations, IN immunization reliably induces higher levels of SARS-CoV-2-specific antibodies in 100% of immunized hamsters.

We further determined post-challenge titers in serum collected from sham control, SC, or IN-immunized hamsters challenged IN with 1 × 10^5^ TCID_50_ of SARS-CoV-2. The antibody responses were slightly elevated from pre-challenge titers in the sham control and SC-immunized 1 × 10^3^ TCID^50^ groups. Similarly, levels of SARS-CoV-2-specific antibodies were higher than their pre-challenge levels in 4 out of 5 hamsters immunized SC with 1 × 10^4^ and 1 × 10^5^ TCID_50_. On the contrary, higher levels of antibodies were detected in all hamsters immunized via the IN route with 1 × 10^5^ TCID_50_. However, because of the variability in the SC-immunized groups, these higher levels of antibodies in the IN-immunized group did not achieve statistical significance (Figure 3B). A similar trend was observed for SARS-CoV-2 spike protein-specific IgG antibody levels on day five post-challenge. However, unlike neutralizing antibody titers, the spike protein-specific IgG levels were significantly higher in hamsters immunized with 1 × 10^4^ and 1 × 10^5^ TCID_50_ by the SC route and 1 × 10^5^ TCID_50_ by the IN route than in sham controls or hamsters immunized SC with 1 × 10^3^ TCID_50_ (Figure 3C). Collectively, these results demonstrate a high variability in antibody responses in hamsters immunized via the SC route. On the other hand, an identical elevated neutralizing antibody response is observed in all hamsters immunized via the IN route. These results also demonstrate that the IN route, rather than the SC route, is an effective route of immunization.

### Intranasal-immunized hamsters control the SARS-CoV-2 replication more effectively than SC-immunized hamsters

**Figure 4:**
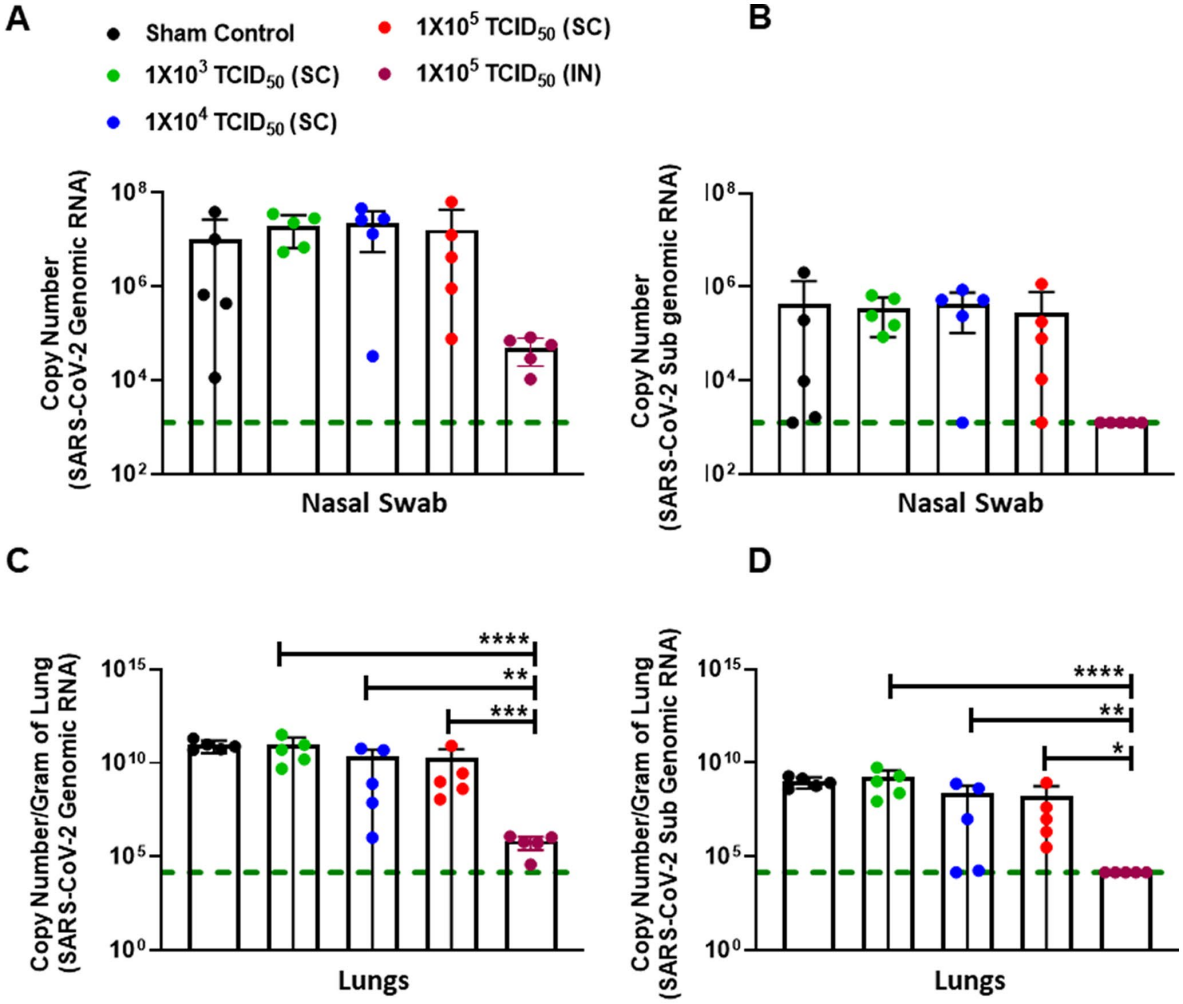
Intranasally-immunized hamsters control the SARS-CoV-2 replication more effectively than subcutaneously immunized hamsters. Sham control and hamsters immunized with the indicated doses of live SARS-CoV-2 by subcutaneous (SC) and intranasal (IN) routes were challenged with 1 ×10^5^ TCID_50_ dose of live SARS-CoV-2 (USA-WA1/2020 isolate) on day 28 post-immunization. Nasal swabs were collected on day three post-challenge and the viral burden was determined by quantitating genomic **(A)** and subgenomic **(B)** SARS-CoV-2 RNA by qRT-PCR. RNA isolated from the lungs on day five post-challenge were quantitated for genomic **(C)** and subgenomic **(D)** copies of SARS-CoV-2 RNA by qRT-PCR. The data were analyzed by ANOVA using *post-hoc* Tukey’s test. ^*^p < 0.05; ^**^p < 0.01; ^***^p < 0.001, ^****^p < 0.0001. The horizontal green dotted line denotes the lower limit of quantitation.

We next investigated whether elevated antibody responses in IN-immunized hamsters effectively controlled SARS-CoV-2 replication after IN challenge. Nasal swabs were taken from all challenged animals on day 3 post-challenge (day 31 post-primary immunization), and the viral load was quantified by determining the genomic and sub-genomic levels of SARS-CoV-2 RNA using qRT-PCR. Genomic RNA is generally considered a measure of the total viral genome contributed by viable and nonviable viral particles. Furthermore, genomic RNA analysis does not discriminate between input virus and virus generated during an ongoing infection. On the other hand, sub-genomic RNA generally represents the replicating virus levels. As observed for the antibody responses, a lot of variability was observed in the genomic and sub-genomic SARS-CoV-2 RNA levels in the sham control and SC immunized groups. On the other hand, consistently lower levels of genomic and sub-genomic SARS-CoV-2 RNA were observed in the group of IN-immunized hamsters (Figures 4A,B). However, despite lower levels in the IN-immunized group, these levels did not achieve statistical significance as compared to the SC-immunized groups when analyzed by one-way ANOVA followed by multiple comparison tests due to considerable variability in the SC-immunized groups.

Next, we determined the genomic and sub-genomic levels of SARS-CoV-2 in the lungs of SC or IN-immunized hamsters 5 days after the challenge (day 33 post-primary immunization). The results showed significantly lower levels of genomic and sub-genomic SARS-CoV-2 RNA in the lungs of hamsters immunized IN with 1 × 10^5^ TCID_50_ than the group of hamsters immunized by SC route and challenged IN with 1 × 10^5^ TCID_50_ of SARS-CoV-2 (Figures 4C,D).

**Figure 5:**
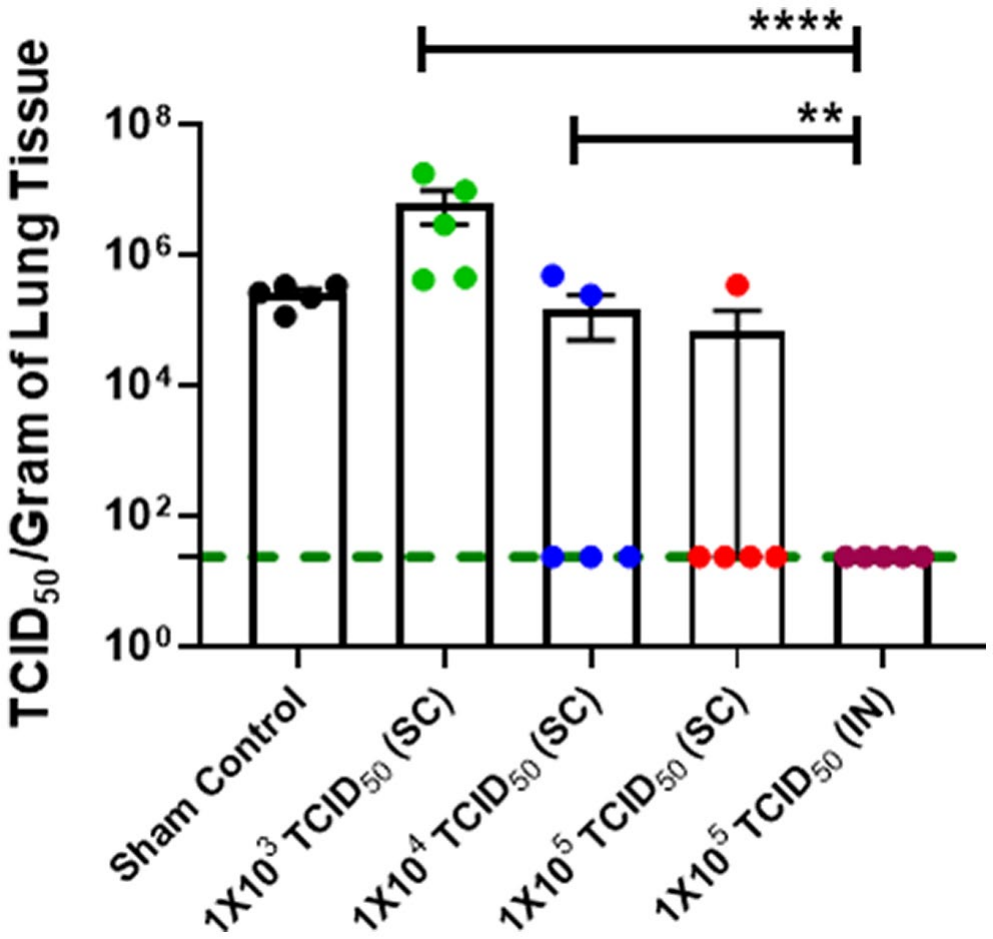
Intranasally-immunized hamsters exhibit low numbers of viable SARS-CoV-2 in the lungs than SC-immunized hamsters. Sham control and hamsters immunized with the indicated doses of live SARS-CoV-2 by subcutaneous (SC) and intranasal (IN) routes were challenged with 1× 10^5^ TCID_50_ tissue culture 50% infectious dose (TCID_50_) of live SARS-CoV-2 (USA-WA1/2020 isolate) on day 28 post-immunization. The lung viral load was determined by TCID_50_ assay. Statistical analysis of the log-transformed data by ANOVA using post-hoc Tukey’s test is shown. ^**^p < 0.01; ^****^p < 0.0001. The horizontal green dotted line denotes the lower limit of quantitation.

We further confirmed the findings from the genomic and sub-genomic RNA analysis by determining the viable viral load in the lungs of immunized and challenged hamsters by determining the TCID_50_, a measure of the viable virus in study samples on day 5 post-challenge. The TCID_50_ data for pulmonary viral burden mirrored SARS-CoV-2 genomic and sub-genomic RNA data (Figures 4C,D) with significantly lower TCID_50_ levels observed for IN-immunized hamsters than hamsters immunized with 1 × 10^3^ and 1 × 10^4^ TCID_50_ doses by the SC route (Figure 5). However, 4/5 hamsters immunized SC with 1 × 10^5^ TCID_50_ showed similar numbers of viable SARS-CoV-2 as observed in the IN-immunized group day five post-challenge. Collectively, these results demonstrate that strong immune responses induced by IN immunization also result in effective clearance of SARS-CoV-2.

### Minimal pathological lesions are observed in the lungs of IN-immunized hamsters following IN challenge with SARS-CoV-2

**Figure 6:**
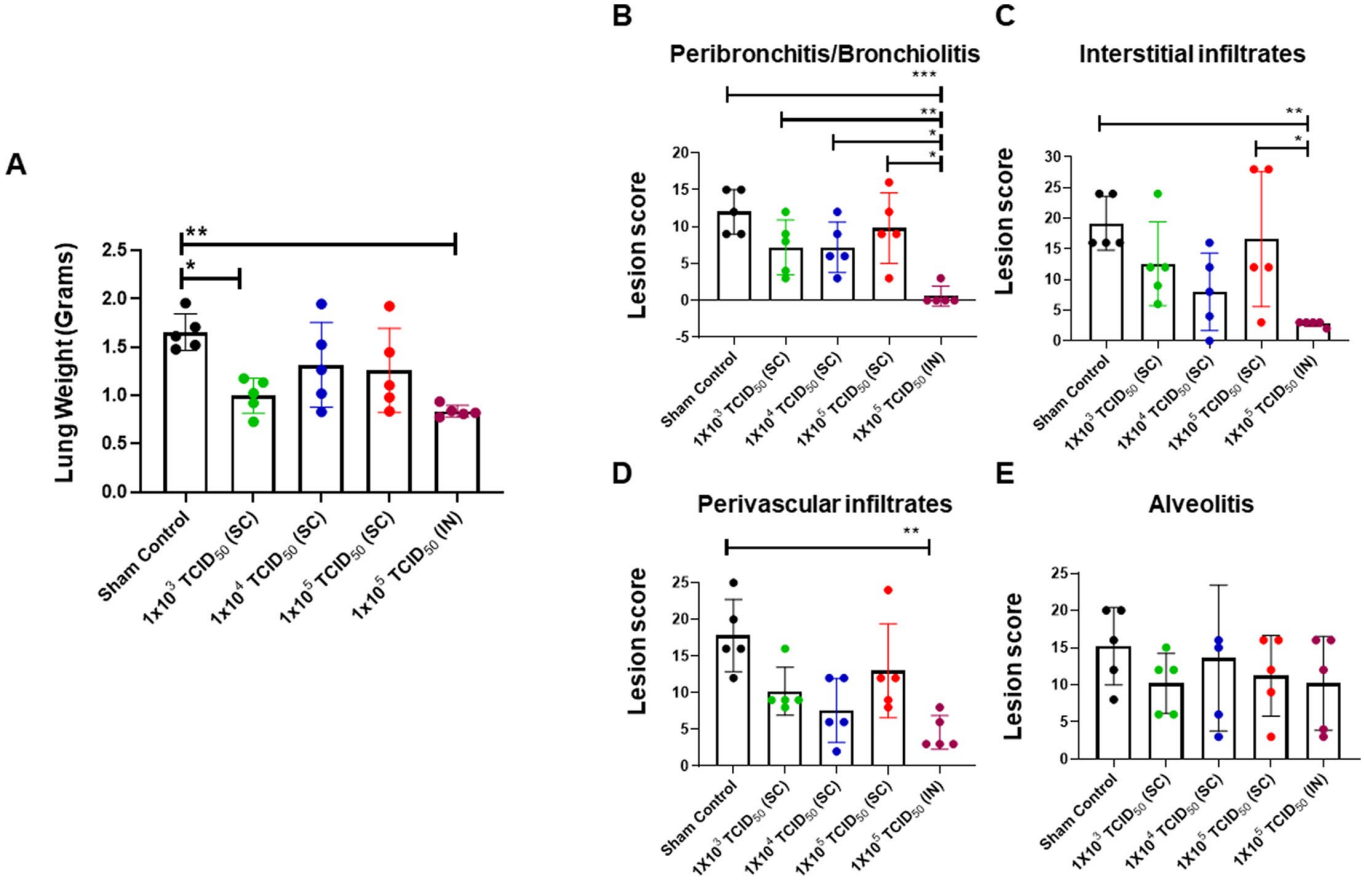
Minimal pathological lesions are observed in the lungs of intranasally-immunized hamsters following intranasal challenge with SARS-CoV-2. Sham control and hamsters immunized with the indicated doses of live SARS-CoV-2 by subcutaneous (SC) and intranasal (IN) routes were challenged with 1 × 10^5^ TCID_50_ dose of live SARS-CoV-2 (USA-WA1/2020 isolate) on day 28 post-immunization. **(A)** The lungs collected on day five post-challenge were weighed. The lungs were fixed in 10% formalin, paraffin embedded, sectioned and stained with hematoxylin and eosin. The sections were scored in a blinded fashion for **(B)** Peribronchitis/bronchiolitis, **(C)** interstitial infiltrates, **(D)** perivascular infiltrates and **(E)** alveolitis. The data were analyzed by ANOVA using *post-hoc* Tukey’s test. ^*^p < 0.05; ^**^p < 0.01, ^***^p < 0.001. The horizontal green dotted line denotes the lower limit of quantitation.

We evaluated the histopathological lesions in the lungs of hamsters that were sham control, SC or IN-immunized and challenged with 1 × 10^5^ TCID_50_ of SARS-CoV-2 via IN administration on day five post-challenge. An increase in lung weight is an indicator of increased inflammation and pulmonary edema in pulmonary infection models. The results showed that the average lung weights were similar in the SC and IN-immunized groups and remained significantly lower compared to the sham control group. However, as observed for the viral load, almost 3/5 hamsters from the SC-immunized groups showed an increased lung weight indicating ongoing inflammation. Consistent with the previous results for the IN-immunized group, no variability in the lung weights were observed, and almost identical low lung weights were recorded for the entire group (Figure 6A). These results indicate that IN-immunization consistently provides uniform protection for all vaccinated subjects.

The lung sections from the control, SC, and IN-immunized hamsters that were challenged IN with 1 × 10^5^ TCID_50_ of SARS-CoV-2 on day 5 post-challenge were further evaluated for histopathological lesions by grading for peribronchiolitis/bronchiolitis, perivasculitis, interstitial infiltrates, and alveolitis. The lungs of the IN-immunized group had significantly lower peribronchiolitis and bronchiolitis compared to the control and SC-immunized groups (Figure 6B). Similarly, the IN-immunized group revealed significantly lower interstitial infiltrates than the sham control and the group immunized SC with 1 × 10^5^ TCID_50_ dose of SARS-CoV-2 (Figure 6C). The perivascular infiltration was similar in all the SC or IN-immunized groups; however, these infiltrates were significantly higher in the sham controls than in the IN-immunized group (Figure 6D). No differences were observed in the magnitude of alveolitis between the groups (Figure 6E). Furthermore, the lungs of IN-immunized hamsters showed the least severe changes consisting of residual inflammation and epithelial hyperplasia, particularly at the bronchioloalveolar duct junctions. These histopathological findings were consistent with the viral loads observed in the SC or IN-immunized hamsters. Collectively, these results further establish that the IN route provides better protection than the SC route of immunization from SARS-CoV-2 infection.

## Discussion

As the COVID-19 pandemic progressed, parenterally delivered vaccines have been proven to provide significant protection from symptomatic infection, hospitalization, and death (Polack et al., 2020; Baden et al., 2021). However, there is a growing interest in developing mucosally-delivered vaccines to further enhance the ease and durability of vaccination (Russell et al., 2020). No vaccine is available for IN immunization. We employed homologous and heterologous immunization and challenge model by utilizing SARS-CoV-2 isolate USA-WA1/2020 for immunization and challenge studies. This strain of SARS-CoV-2 was isolated from a patient on January 19, 2020 in Washington state. We compared the efficacy of SC and IN routes of immunizations against an IN challenge. Our results demonstrate that the IN route is superior to the SC route of immunization in protecting against a respiratory infection caused by SARS-CoV-2.

Like humans, the spike protein of SARS-CoV-2 binds efficiently with hamster ACE2 receptor and thus hamsters can be easily infected with the SARS-CoV-2 virus (Liu et al., 2021). The disease symptoms in Syrian hamsters such as weight loss, lung damage, and high viral loads respiratory tracts and pathological lesions are similar to those observed in human COVID-19 disease (Chan et al., 2020). The similarity between hamsters and human respiratory system makes them a valuable animal model for studying the aerosol spread of the SARS-CoV-2 virus and the pathogenesis of COVD-19. Similar to humans, SARS-CoV-2 can be transmitted through aerosols between hamsters (Braxton et al., 2021). On the other hand, K18-transgenic mice that are commonly used in animal studies are genetically engineered to express human ACE2 receptors. Naturally, K18 mice are not the natural host for SARS-CoV-2 and therefore, their response also differs from human SARS-CoV-2 infection. Since hamsters are a more physiologically relevant model for studying the COVID-19 disease, we used this model for immunization and challenge studies.

In the present study, we used the term “immunization” for the primary infection of SARS-CoV-2 administered via either the SC or IN routes and “challenge” as the secondary infection by the same strain of the virus administered via the IN route. Thus, the study design using the IN route for immunization and challenge mimicked the features of natural primary infection and secondary re-infection with SARS-CoV-2. Individuals naturally infected with SARS-CoV-2 have a reduced risk of severe disease, and a majority show milder symptoms following subsequent re-infections (Deng et al., 2023). Our results also demonstrate similar features. Hamsters receiving the primary and secondary infections via the IN route did not experience weight loss, mounted a higher neutralizing antibody response, and quickly cleared the SARS-CoV-2 virus with minimal to no lung damage.

Several skin conditions have been associated with COVID-19. These conditions are mostly observed during the post-COVID phase (Tan et al., 2021). However, no evidence is available to suggest the transmission of SARS-CoV-2 can occur via the skin. The outermost layers of the skin, consisting of keratinocytes, do not express the ACE2 receptor required for the attachment and entry of the SARS-CoV-2 virus. However, the skin cells between the dermis and epidermis, particularly the stratum basale, sweat glands, and blood endothelial cells, express the ACE2 receptor and can support the replication of SARS-CoV-2. Nonetheless, a skin integrity breach is necessary for the virus to gain access to these basal layers. It has been reported that in mice with impaired skin barrier function, the SARS-CoV-2 enters the basal layers of the skin, replicates locally in the skin, and subsequently disseminates to the lungs (Xu et al., 2021, 2022). Since no evidence is available to indicate that SARS-CoV-2 is transmitted naturally through the skin, the SC immunization approach used in this study, followed by an IN challenge, does not represent a natural infection and re-infection model. Instead, it mimics the current vaccination approaches used to prevent COVID-19 and the subsequent SARS-CoV-2 infection acquired through the respiratory tract in vaccinated individuals.

The pattern of body weight loss in the SC-immunized groups indicated that the initial response was milder than that observed in the IN-immunization group. However, it is worth noting that we used a very high dose of 1 × 10^5^ TCID_50_ of SARS-CoV-2 for IN immunizations. We speculate that a lower dose of IN immunization could result in a milder response while maintaining the same magnitude of immune response and protective efficacy. One intriguing feature we noticed was that the post-challenge weight loss was minimal in hamsters immunized with a 1 × 10^3^ TCID_50_ dose by the SC route, despite having low neutralizing and spike protein-specific antibody titers compared to other SC immunized groups. Furthermore, their viral loads were similar to other SC-immunized groups. However, their lung weights and severity of lung lesions were lower than the SC-immunized groups and more similar to the IN-immunized groups. To our knowledge, this is the first study to report the effects of live SARS-CoV-2 delivered by the SC route in the hamster model. Therefore, the pattern of dissemination of the virus to other organs, especially the lungs, following an SC route of administration of SARS-CoV-2 is unknown. Studies have shown that SARS-CoV-2 binds to human ACE2 expressed by its target cells and uses it as a functional receptor to enter cells (Hoffmann et al., 2020; Walls et al., 2020). In hamsters and other species, ACE2 expression and tissue distribution appear to dictate patterns of SARS-CoV-2 infection (Suresh et al., 2021; Lean et al., 2022; Tomris et al., 2022). ACE2 expression in the respiratory and gastrointestinal tract likely predominates in initial interactions with the virus via the IN route in hamsters (Suresh et al., 2021; Tomris et al., 2022). It would be of great interest to evaluate virus levels in different organs when the SARS-CoV-2 virus is administered via the SC route to determine the pattern of dissemination of the virus, especially when varying doses of the virus are used. An effective lower dose of the virus delivered to the lung and nasal mucosa via the SC route of immunization may explain the differences observed between the lower and higher SC immunization doses in terms of body weight loss and lung damage in response to a subsequent respiratory infection.

Other studies have shown that, following a primary lung infection, significant immune memory and protection from subsequent SARS-CoV-2 infection is developed (Brustolin et al., 2021; Horiuchi et al., 2021; Singh et al., 2021, 2022; Yinda et al., 2021; Field et al., 2022). Primary infection results in elevated levels of neutralizing antibodies and IgG–IgA and the generation of virus-specific T and B cells that are preserved as memory cells for 4–6 months. Further, there are reports showing mucosal (oral or IN) delivered vaccines similarly induce a robust immune response and provide protection against infection and reduce subsequent transmission (Johnson et al., 2022; Langel et al., 2022).

We observed a stronger pre-challenge immune response in IN-immunized hamsters as measured by the presence of neutralizing antibodies at 28 days post-immunization that resulted in substantial protection from a subsequent IN live SARS CoV-2 challenge. One hundred percent of the IN-immunized hamsters exhibited reduced body weight loss, reduced lung viral load and reduced lung pathology. In contrast, the SC-immunized groups showed low levels of pre-challenge neutralizing antibody responses. However, increased levels of post-challenge serum-neutralizing antibodies in SC-immunized groups indicated that immune responses are amplified following a subsequent IN challenge with the homologous SARS CoV-2. However, there was a great variability observed in terms of pre-and post-challenge antibody responses as well as all other parameters investigated in the SC-immunized group. On the other hand, 100% of animals in the IN-immunized group responded identically to both the primary vaccination and the subsequent challenge. These results further substantiate the notion that the availability of IN immunization platform will achieve herd immunity reliably against COVID-19 and other respiratory pathogens.

A study compared the protective efficacy of mRNA, an adenovirus-vectored and a live attenuated COVID-19 vaccine by using either a single IM immunization or an initial priming by IM immunization followed by an IN booster in Syrian hamsters. They reported that all vaccination strategies rendered some degree of protection against an IN challenge with SARS-CoV-2 Delta variant. However, IM priming followed by the IN booster with the live attenuated COVID-19 vaccine induced a robust and superior mucosal immune response than the hamsters receiving IM immunizations only (Nouailles et al., 2023). The results from our study are in agreement with these findings and demonstrate that the administration of live virus by the SC route facilitates the development of a systemic neutralizing antibody response similar to that observed for the IN-immunized hamsters. However, higher viral loads in the nasal mucosa and lungs and the extent of lung damage observed following the IN challenge indicate that the SC route of immunization is inefficient at inducing a protective mucosal immune response achieved through the IN route of immunization. Although the mucosal immune responses were not determined in the current study, these results point to the fact that the systemic antibody responses following parenteral immunization are not an accurate measure of the protective efficacy of the vaccines at the mucosal surfaces. Therefore, effective measures that can determine the immune responses at the mucosal surfaces need to be developed to evaluate the effectiveness of a vaccine administered through a parenteral route for protection against respiratory infections, including COVID-19.

While the current study was simple in design and performed a basic characterization of the differences between SC and IN immunizations with live SARS-CoV-2, there were some limitations. The study was designed to understand whether immunization via SC compared to IN route of delivery of live SARS-CoV-2 could reduce the severity of infection against a subsequent IN SARS-CoV-2 challenge. Although we analyzed neutralizing antibody responses, we did not measure SARS-CoV-2 spike protein-specific mucosal IgA or T-cell responses, which play a significant role in protection against SARS-CoV-2 (Baker et al., 2022). While we matched the highest SC dose to the IN dose, a more robust immune response may have been achieved with an even higher SC dose. This dose differential is commonly observed with IN versus systemic drug administration, where less is required by the pulmonary route to achieve the same pharmacological effect in the lung. Furthermore, we used only a single primary SC and IN immunization to induce immunity, whereas many available COVID-19 vaccines use two or more doses. Therefore, our future studies will focus on addressing these shortcomings to improve the protective efficacy of the IN route of vaccination against COVID-19. However, the use of live SARS-CoV-2 virus is most similar to vaccines such as the Johnson & Johnson vaccine, where only a single dose is utilized. More importantly, the immune response to a single IN immunization dose indicates that this strategy could simplify the vaccination of large populations by allowing immunity to be achieved with a single vaccination.

## Conclusion

The findings of this study demonstrate that immunization through the IN route is more effective than SC route in safeguarding against respiratory infection caused by SARS-CoV-2. These results further emphasize the benefits of IN immunization in eliciting a strong and dependable immune response to live SARS-CoV-2, thus advocating for a switch to IN immunization strategies. Additionally, this study underscores the potential of mucosal immunization approaches in triggering a consistently strong immune response and offering superior protection compared to other methods. In summary, this study demonstrates that the route of primary immunization plays a critical role in determining the severity of a subsequent respiratory infection caused by SARS-CoV-2 and provides evidence in favor of IN immunization as a preferred strategy for combating COVID-19 caused by SARS-CoV-2.

## Data availability statement

The datasets presented in this article are not readily available because all the data generated are presented in this manuscript. Requests to access the datasets should be directed to SA, Salomon_Amar@nymc.edu.

## Ethics statement

All immunization and challenge studies were performed in an animal biosafety level 3 facility at the Lovelace Biomedical Research Institute (LBRI), Albuquerque, New Mexico. All animal studies were conducted according to the protocols approved by the Institutional Animal Care and Use Committee (IACUC).

## Author contributions

EB and DR carried out the experiments and data analysis. CB analyzed the data and drafted the manuscript. AK and SA conceived, coordinated the research and finalized the manuscript. All authors contributed to the article and approved the submitted version.

## Funding

This work was supported by internal funds from Lovelace Biomedical Research Institute and New York Medical College and a grant from CVC Credit Partners.

## Conflict of interest

The authors declare that the research was conducted in the absence of any commercial or financial relationships that could be construed as a potential conflict of interest.

## Publisher’s note

All claims expressed in this article are solely those of the authors and do not necessarily represent those of their affiliated organizations, or those of the publisher, the editors and the reviewers. Any product that may be evaluated in this article, or claim that may be made by its manufacturer, is not guaranteed or endorsed by the publisher.
